# Using spectral derivatives to remove the influence of hair on optical images of the static absorbing properties of tissue-like turbid media

**DOI:** 10.1117/1.NPh.11.2.025002

**Published:** 2024-04-26

**Authors:** Jeremy C. Hebden, Gianna Forrester, Haoyang Zhang, Danica M. Pacis

**Affiliations:** University College London, Department of Medical Physics & Biomedical Engineering, London, United Kingdom

**Keywords:** near-infrared spectroscopy, diffuse optical imaging, wavelength modulation, absolute imaging

## Abstract

**Significance:**

Although measurements of near-infrared light diffusely reflected from the head and other biological tissues are commonly used to generate images revealing changes in the concentrations of oxy- and deoxy-hemoglobin, static imaging of absolute concentrations has been inhibited by the unknown and variable coupling between the optical probe and the skin, to which hair is often a significant contributor. Measurements of spectral derivatives provide a means of overcoming this shortcoming.

**Aim:**

The aim is to demonstrate experimentally that measurements of the derivative of the attenuation of the detected signal with respect to wavelength can be used to achieve images that are immune to the spatial variation of hair on the surface. The objective is to generate topographic images representative of static absorbing properties rather than retrieving absolute optical coefficients, which requires a tomographic approach.

**Approach:**

The surface of a tissue-equivalent phantom, containing targets with different concentrations of absorbing dye, was coated with a layer of dark hair. The phantom was then imaged using a broadband source and spectrometer, and prior knowledge of the absorbing characteristics of the dye and of melanin was used to acquire separate images of each.

**Results:**

The targets within the phantom are revealed with remarkable clarity, although a nonlinear relationship between the target contrast and absorption was observed. This nonlinear behavior was confirmed and explained using a Monte Carlo model of light propagation in a slab of similar absorbing properties.

**Conclusions:**

A spectral derivative approach could be an effective tool for *in vivo* topographic imaging of the static optical properties of the brain and other tissues, avoiding the deleterious effects of hair.

## 1 Introduction

Medical applications of diffuse optical techniques have been driven by their ability to monitor tissue oxygenation and metabolism noninvasively, due to the significant differences between the characteristic absorption of oxy- and deoxy-hemoglobin (${\mathrm{HbO}}_{2}$ and Hb) and of cytochrome-c-oxidase at near-infrared (NIR) wavelengths. A primary application is the *in vivo* study of the brain, including cerebral oximetry using NIR spectroscopy (NIRS) and functional brain imaging (known as fNIRS^1^). Light that propagates through the skin and skull to the brain is scattered back to the surface, carrying with it information about the concentrations of ${\mathrm{HbO}}_{2}$ and Hb in the cortical regions underlying each source-detector pair, which are tightly coupled to the local neuronal metabolic demand. Meanwhile, fNIRS commonly involves distributing multiple sources and detectors over a large region of the scalp and observing concentration changes at multiple discrete locations in response to brain activity. Such measurements can be converted into topographical maps or used to generate images by applying an image reconstruction method (known as diffuse optical tomography, DOT^2^).

Despite considerable progress in the development and medical applications of both fNIRS and DOT technology, measurements of diffuse reflectance/transmittance on which they both rely are highly sensitive to the uncertain and variable coupling of light into and out of the skin surface. This sensitivity largely inhibits the routine derivation of absolute concentrations of chromophores (${\mathrm{HbO}}_{2}$ and Hb) *in vivo*, and NIRS and DOT measurements are commonly contaminated by artifacts due to large and random changes in coupling due to relative motion between the tissue and the optical probe. Consequently, most NIRS and DOT systems rely on the measurement of changes in signal under conditions in which it can be assumed that the unknown coupling has remained constant, and the changes in signal are used to derive changes in chromophore concentrations. For brain imaging applications, a major contributor to unknown coupling is the presence of hair.

A new technique, known as wavelength-modulated NIRS (WM-NIRS), was recently proposed; it measures the derivative of the attenuation of the detected signal with respect to wavelength.^3^^,^^4^ The theory of WM-NIRS is briefly summarized in Sec. 2. The objective of the work described here is to explore whether, as previously hypothesized, a wavelength modulation approach can be used to produce diffuse optical images that are immune to the variability in surface coupling due to hair. This was investigated using a solid tissue-equivalent phantom, containing eight targets of contrasting absorption, and data acquired using a broadband source and optical spectrometer. Also reported below are the results of a Monte Carlo simulation of the experiment measurements performed to confirm and interpret the observed nonlinear relationship between the target contrast and absorption.

## 2 Wavelength-Modulated NIRS

A typical NIRS measurement involves placing two optical fibers in contact with the surface of the interrogated tissue, separated by a distance d, where one fiber delivers light to the surface and the other transmits light emitted from the surface to a detector. For a homogeneous tissue, the detected intensity I is described by the modified Beer–Lambert law as follows:

$$I=kI_{0}e^{-\mu_{a}\beta d-G},$$ (1)

where $\mu_{a}$ is the tissue absorption coefficient, β is the differential pathlength factor (DPF), G is an unknown scatter-dependent geometric factor, $I_{0}$ is the source intensity, and k is the (unknown and highly variable) coupling efficiency. The DPF is equal to the average pathlength of detected photons divided by d, and the factor $e^{-G}$ describes the reduction in detected intensity due to scatter. The value of $\mu_{a}$ depends on the concentrations $c_{n}$ of the constituent chromophores as follows:

$$\mu_{a}=\underset{n}{\sum }\varepsilon_{n}c_{n},$$ (2)

where n is the number of chromophores and $\varepsilon_{n}$ are their molar extinction coefficients. Because k and G are unknown, the applications of NIRS and DOT are mostly restricted to observing changes in chromophore concentration and/or oxygenation, under conditions in which it is assumed that k and G remain constant. Although methods have been explored for identifying and/or eliminating artifacts due to variable coupling in recorded data,^5^^–^^8^ some investigators have suggested alternative types of optical measurement that are less sensitive to coupling variability. Specifically, Dehghani et al.^9^^,^^10^ and Pucci et al.^11^ noted the insensitivity of spectral derivatives to coupling. WM-NIRS measures the spectral derivative directly,^3^^,^^4^ for example, by modulating the source wavelength at a fixed frequency. Any variation in tissue absorption over the wavelength range will result in a small modulation in detected intensity, which can be measured using lock-in or Fourier methods.

Using Eq. (1), the attenuation A of light between the source and detector are expressed as

$$A=-\ln(\frac{I}{I_{0}})=\mu_{a}\beta d+G-\ln(k).$$ (3)

Differentiating this equation with respect to wavelength λ yields

$$\frac{\partial A}{\partial \lambda }=\frac{1}{I_{0}}\frac{\partial I_{0}}{\partial \lambda }-\frac{1}{I}\frac{\partial I}{\partial \lambda }=d(\beta \frac{\partial \mu_{a}}{\partial \lambda }+\frac{\partial \beta }{\partial \lambda }\mu_{a})+\frac{\partial G}{\partial \lambda }-\frac{1}{k}\frac{\partial k}{\partial \lambda }.$$ (4)

The hypothesized immunity of WM-NIRS to unknown and variable coupling stems from the fact that the rate of change of attenuation A with wavelength ∂A/∂λ is independent of the absolute magnitude of the measured intensity I and an assumption that coupling is wavelength independent (i.e., ∂k/∂λ=0).

In principle, the immunity to unknown coupling losses enables measurements of the attenuation gradient to be used to estimate absolute values of optical properties. This was recently explored using a series of experiments on turbid samples and models of light transport in turbid media.^4^ These studies empirically observed that, for homogenous media, Eq. (4) is simplified as

$$\frac{\partial A}{\partial \lambda }\approx d\langle \beta \rangle \sum_{n}\frac{\partial \varepsilon_{n}}{\partial \lambda }c_{n}-\alpha \lambda^{-p},$$ (5)

where α and p are positive constants and ⟨β⟩ is the mean value of the DPF calculated over the full range of wavelengths. The term $\alpha \lambda^{-p}$ was introduced to accommodate the influence of the wavelength dependence of the transport scattering coefficient $\mu_{s}^{\prime }$. In practice, the variation of DPF in the human head is relatively small and has a typical value of ∼5.^12^ Most surprisingly, Eq. (5) was found to be highly robust to changes in measurement geometry providing that such changes are reflected in the estimate of the mean DPF (i.e., no independent assessment of the scattering-dependent geometric parameter G in the modified Beer–Lambert law is required).^4^

Given that d, ⟨β⟩, and $\partial \varepsilon_{n}/\partial \lambda$ are either known or can be reliably estimated, measurements of ∂A/∂λ at multiple discrete wavelengths can be used to derive the concentrations $c_{n}$ and the constants α and p by solving Eq. (5) as a matrix equation or using a minimum norm least-squares approach. Experimental and modeling results suggest that chromophore concentrations in homogeneous tissue-like turbid media could be estimated with a typical accuracy of better than 10%,^4^ comparable to the accuracy achieved by Dehghani et al.^9^ using spectral derivatives estimated from simulated tissue data. The number of discrete wavelengths at which ∂A/∂λ should be sampled should not be less than the number of unknowns in Eq. (5). Furthermore, to ensure a unique solution, it is important that those discrete wavelengths correspond to spectral regions where the extinction coefficient derivatives $\partial \varepsilon_{n}/\partial \lambda$ are most significant and are distinct from each other.^3^

## 3 Methods

### 3.1 Phantom Design

**Fig. 1:**
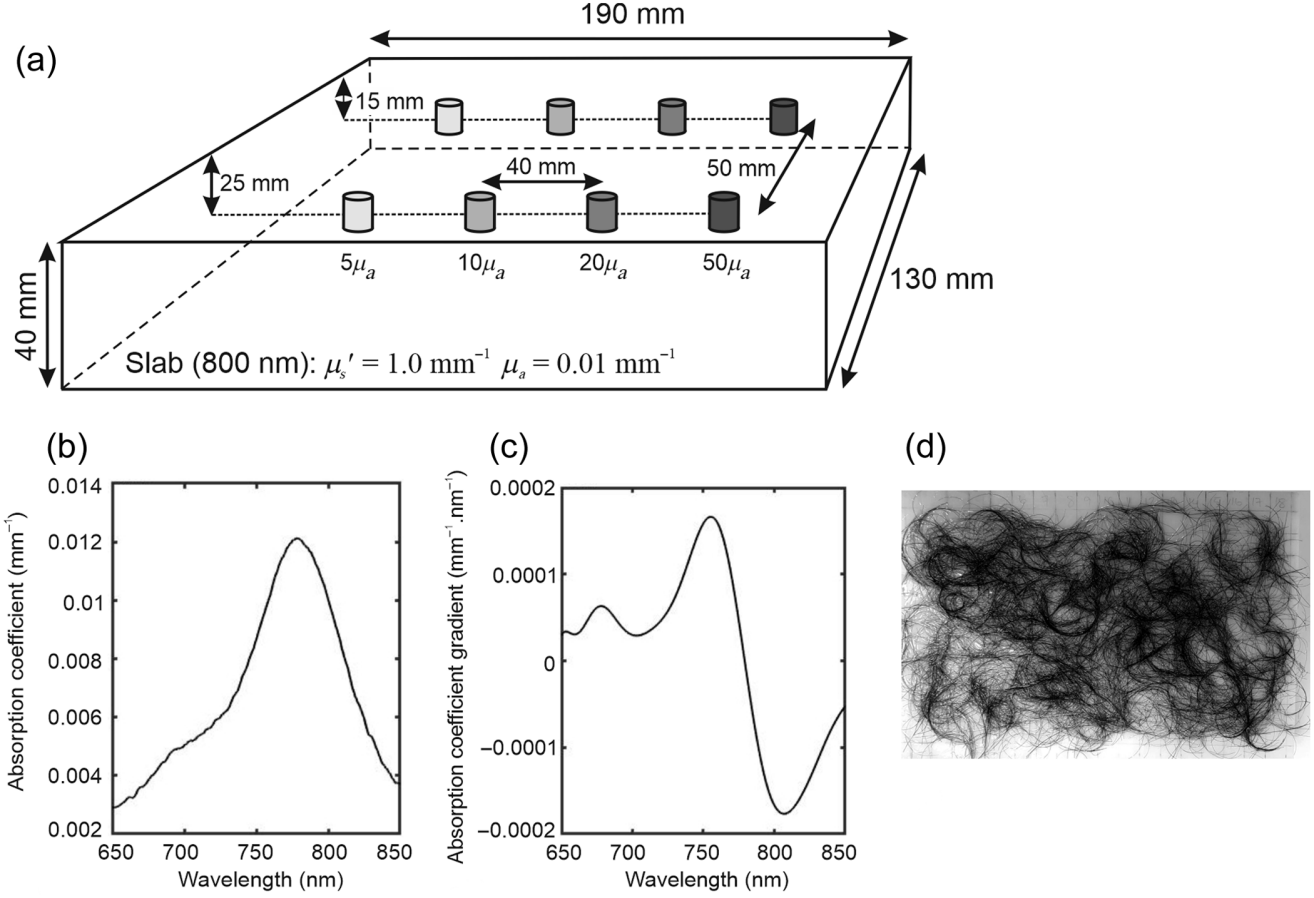
(a) Schematic of a tissue-equivalent phantom containing eight targets with four contrasting absorption coefficients (5, 10, 20, and 50 times background) and at two different depths (15 and 25 mm) below the surface. (b) Absorption coefficient of the phantom block. (c) First derivative of the absorption coefficient spectrum. (d) Image of the top surface of the phantom coated with a heterogeneous layer of dark hair from six adult volunteers, bonded with a 0.8-mm layer of translucent silicone rubber.

To explore whether a wavelength modulation approach can be used to produce images that are immune to the variability in surface coupling due to hair, a solid phantom was employed with tissue-like scattering and absorbing properties at NIR wavelengths. The phantom, described in full elsewhere,^13^ consists of a solid rectangular block (dimensions of 130×190×40  mm) cast from polyester resin mixed with titanium dioxide particles and NIR dye (S101753 manufactured by ICI, London, UK), as illustrated in Fig. 1(a). The transport scatter coefficient is approximately $\mu_{s}^{\prime }=1.0\;{\mathrm{mm}}^{-1}$ at a wavelength λ=800  nm, and the absorption coefficient has a wavelength dependence, as shown in Fig. 1(b). Embedded within the block are two rows of four cylindrical targets (length of 10 mm and diameter of 10 mm). The targets, which have the same scattering properties as the surrounding block, have absorption coefficients 5, 10, 20, and 50 times that of the block, and their centers are embedded at two different depths from the surface: 15 and 25 mm. The spectral gradient of the dye absorption coefficient within the block, displayed in Fig. 1(c), has a maximum of around $\partial \mu_{a}/\partial \lambda \approx 0.00017\;{\mathrm{mm}}^{-1}{\cdot \mathrm{nm}}^{-1}$ at a wavelength λ=756  nm and a minimum of about $\partial \mu_{a}/\partial \lambda \approx -0.00018\;{\mathrm{mm}}^{-1}\cdot {\mathrm{nm}}^{-1}$ at a wavelength λ=808  nm. This is consistent with the gradient values expected for *in vivo* brain tissues, which are within the range $\pm 0.0002\;{\mathrm{mm}}^{-1}{\cdot \mathrm{nm}}^{-1}$ over the NIR wavelength interval 740 to 860 nm.^4^ As shown in Fig. 1(d), the top surface of the block was coated with dark hair from six adult volunteers and bonded with a 0.8-mm layer of translucent silicone rubber.

### 3.2 Spectrometer Measurements

**Fig. 2:**
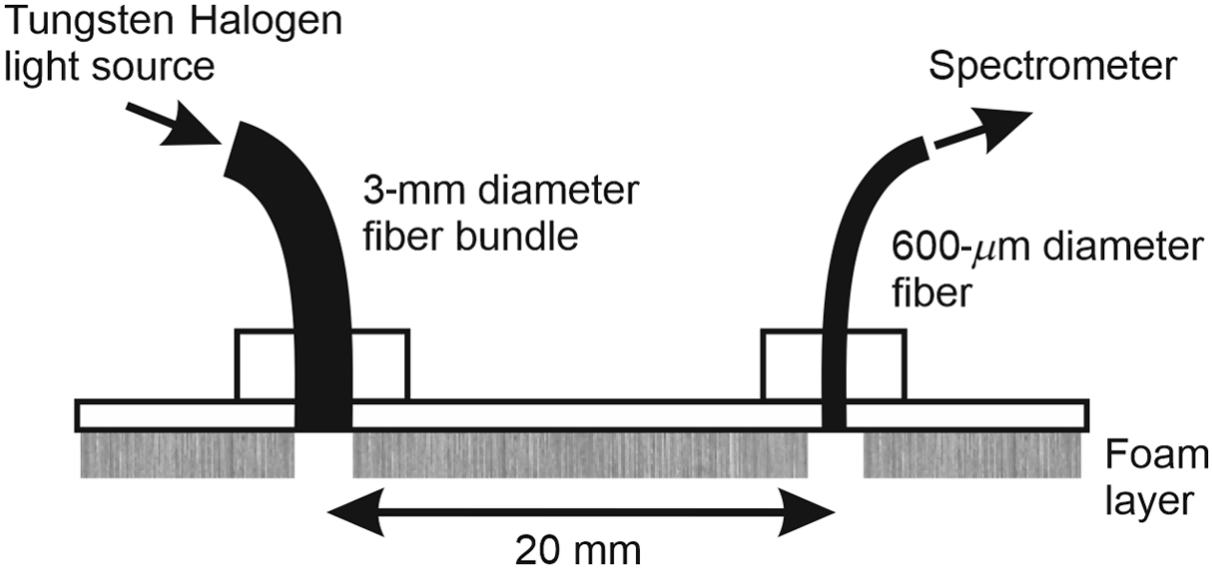
Probe used to acquire spectrometer measurements on the surface of the phantom. It supports a fiber bundle connected to a tungsten halogen light source and a fiber coupled to the spectrometer, located 20 mm apart.

The quantity ∂A/∂λ in Eq. (4) can be derived by estimating the gradient of the logarithm of the measured intensity as a function of wavelength. Such measurements were acquired on the phantom using an optical spectrometer and the simple probe illustrated in Fig. 2. It consists of two optical fiber connectors mounted on a rigid plastic plate such that the attached fibers are secured 20 mm apart. The lower surface of the probe is coated with a 5-mm thick layer of light-absorbing foam, with 5-mm diameter holes directly below each optical fiber. A spectrometer (Ocean Insight HR2000+ CG) was coupled to one of the two connectors via a 600-μm diameter fiber, and a broadband light source (Ocean Insight Tungsten-Halogen HL-2000-HP) was coupled to the other via a 3-mm diameter fiber bundle. The source delivers an output power of the order of 10  μW/nm at ∼800  nm, and the spectrometer has a spectral resolution of ∼1  nm.

Before adding the layer of hair, the phantom was manually scanned by placing the probe at 150 discrete locations on the surface: 10 horizontal rows, 1 cm apart, were scanned, with each row consisting of 15 samples at intervals of 1 cm. A spectrum was recorded for 15 s at each location. Meanwhile, a source spectrum was acquired separately by coupling the source directly to the spectrometer. After the subtraction of background noise, a 12^th^-order polynomial was fitted to the logarithm of each spectrum between wavelengths of 700 and 840 nm, sufficient to account for subtle features in the broadband spectrum of the source. The gradient of each log spectrum at any wavelength within the fitted interval could then be estimated using the parameters of the corresponding polynomial. The phantom was then scanned again after the layer of hair was applied to the surface, as shown in Fig. 1(d). Spectra were recorded for 30 s per location, and similar processing of the acquired data was performed.

The possibility of “light piping” through the layer of silicone rubber and hair was briefly explored by placing the same layer in contact with a reflective surface such that light could only reach the detector via the layer. Measurements using the probe were below the noise level of the spectrometer, confirming that the light piping effect is very likely suppressed due to the very high absorption by hair over the 20-mm direct path between the source and detector.

## 4 Imaging Results

### 4.1 Images Representing Variation in Attenuation and Attenuation Gradient

#### 4.1.1 Phantom without hair layer

**Fig. 3:**
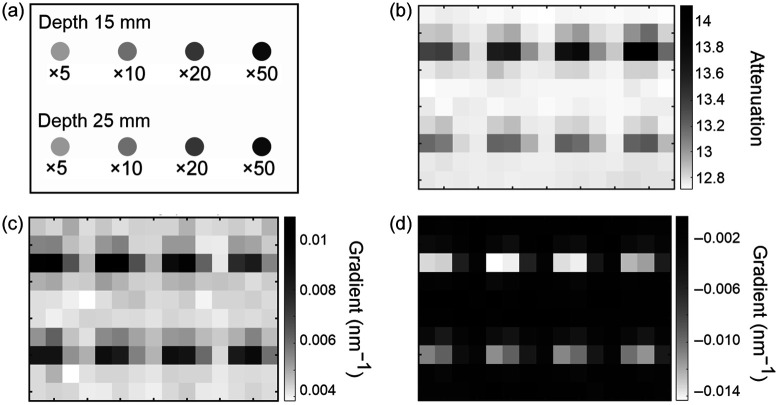
Images of the phantom without the hair layer, generated using spectrometer measurements. (a) Locations and relative absorptions of eight embedded targets within the imaged area (150  mm×100  mm) of the phantom; (b) image obtained using the measured attenuation (log of the integrated signal 700 to 840 nm); (c) image obtained using the measured attenuation gradient at a wavelength of 760 nm; (d) image obtained using the measured attenuation gradient at a wavelength of 800 nm.

Using the spectra recorded on the phantom without the layer of hair, an image was generated representing the spatial variation in attenuation [parameter A in Eq. (3)] as the probe was translated across the surface. Values of attenuation for each spectrum were generated by calculating the natural logarithm of the ratio between the signal integrated over the 700 to 840 nm range and the signal acquired from the source spectrum integrated over the same range. The result is shown in Fig. 3(b). The locations of the eight targets are easily identified as regions of high attenuation, and as expected, the highest absorbing targets closest to the surface attenuate the greatest. The relative absorption and true locations of the targets are illustrated in Fig. 3(a).

**Fig. 4:**
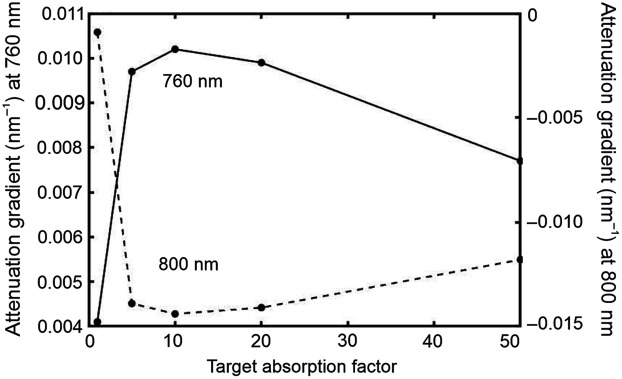
Values of the attenuation gradient measured at 760 nm (solid line and left vertical axis) and 800 nm (dashed line and right vertical axis) on the phantom without the hair layer at locations closest to each of the four embedded targets at a depth of 15 mm, plotted as a function of the target absorption factor.

Images representing the spatial variation in the first derivative of attenuation with respect to wavelength [parameter ∂A/∂λ in Eq. (4)] at selected wavelengths were then generated. These values were calculated by subtracting the gradient of the log of each spectrum from the corresponding gradient of the log of the source spectrum. The results for λ=760  nm and λ=800  nm are shown in Figs. 3(c) and 3(d), respectively. Consistent with the spectral gradient of the dye absorption in Fig. 1(c), the λ=760-nm image reveals the eight targets as regions of higher (increasingly positive) gradient relative to the background, whereas the λ=800  nm reveals them as regions of lower (increasingly negative) gradient. However, the contrast exhibited by the targets clearly does not correlate positively with relative absorption: the targets with the highest intrinsic absorption exhibit the lowest contrast. This is illustrated in Fig. 4, which shows the values of the attenuation gradient measured at 760 and 800 nm on the phantom without the hair layer at the locations closest to each of the four embedded targets at a depth of 15 mm below the surface, plotted as a function of the target absorption factor.

This unexpected phenomenon is explored in Sec. 5.

#### 4.1.2 Phantom with hair layer

**Fig. 5:**
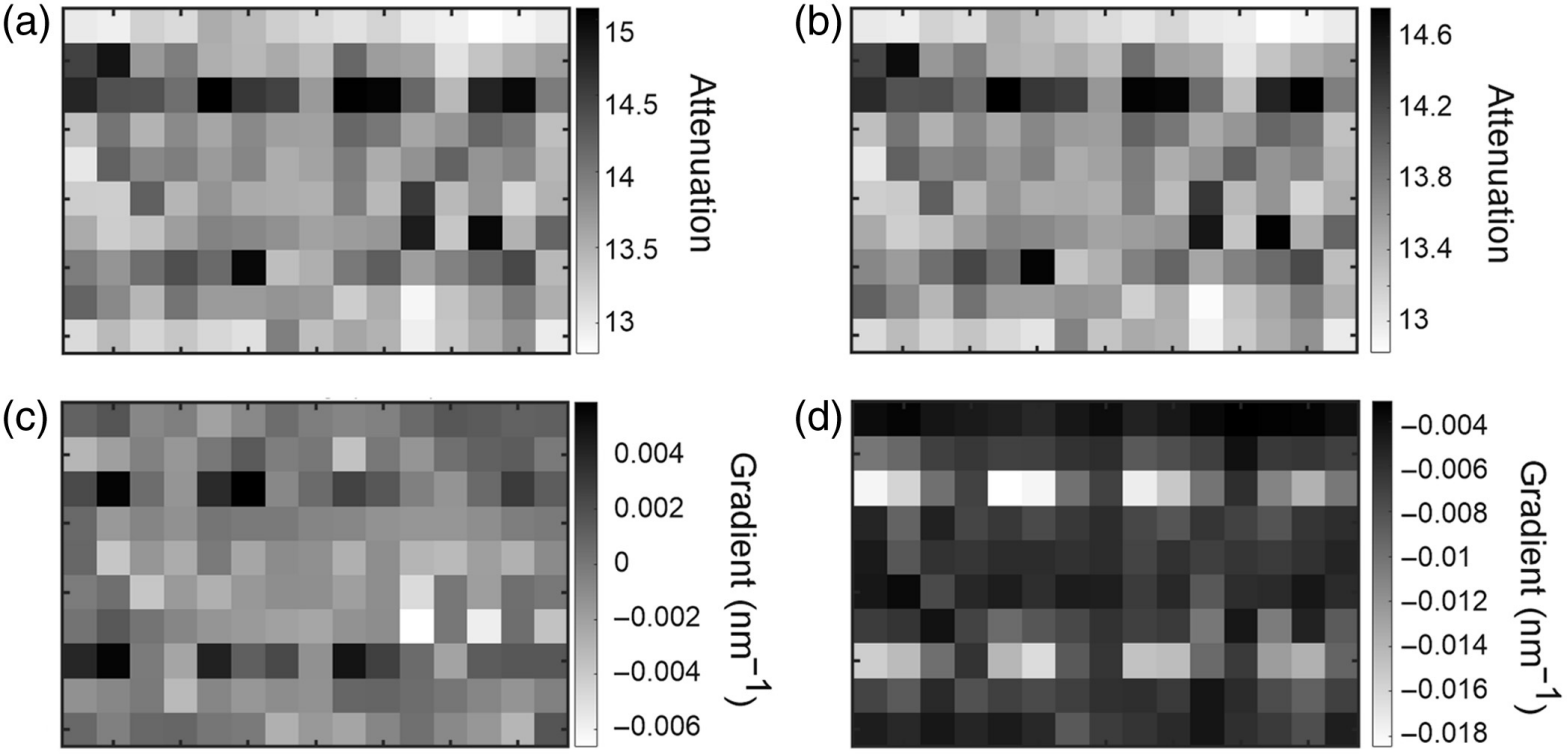
Images of the phantom with the hair layer, generated using spectrometer measurements. (a) Image obtained using the measured attenuation at 760 nm; (b) image obtained using the measured attenuation at 800 nm; (c) image obtained using the measured attenuation gradient at a wavelength of 760 nm; (d) image obtained using the measured attenuation gradient at a wavelength of 800 nm.

Figures 5(a) and 5(b) show the images representing the variation in attenuation as measured across the surface of the phantom with the layer of hair, derived at wavelengths of 760 and 800 nm, respectively. The values were obtained by first subtracting polynomial fits to the logarithm of each spectrum from a polynomial fit to the logarithm of the source spectrum. The two images are qualitatively very similar to each other, and as anticipated, the contrast is strongly influenced by the irregular distribution of hair on the surface. It is impossible to confidently identify the locations of the four deeper targets.

Figures 5(c) and 5(d) show images of the attenuation gradient estimated at the same two wavelengths. The visibility of the targets is evidently improved compared with the attenuation images, and there are similarities with the corresponding images obtained on the phantom without hair [Figs. 3(c) and 3(d)]. However, similar to the attenuation images, the gradient images also exhibit a degree of variation uncorrelated with the target’s location (e.g., across the middle two rows), much larger than that expected due to random noise in the data and most likely due to hair. Repeated measurements at a single location indicated a random variation of less than 0.01 in attenuation and less than $0.0001\;{\mathrm{nm}}^{-1}$ in the gradient.

### 4.2 Images Representing Variation in Absorber Concentrations

**Fig. 6:**
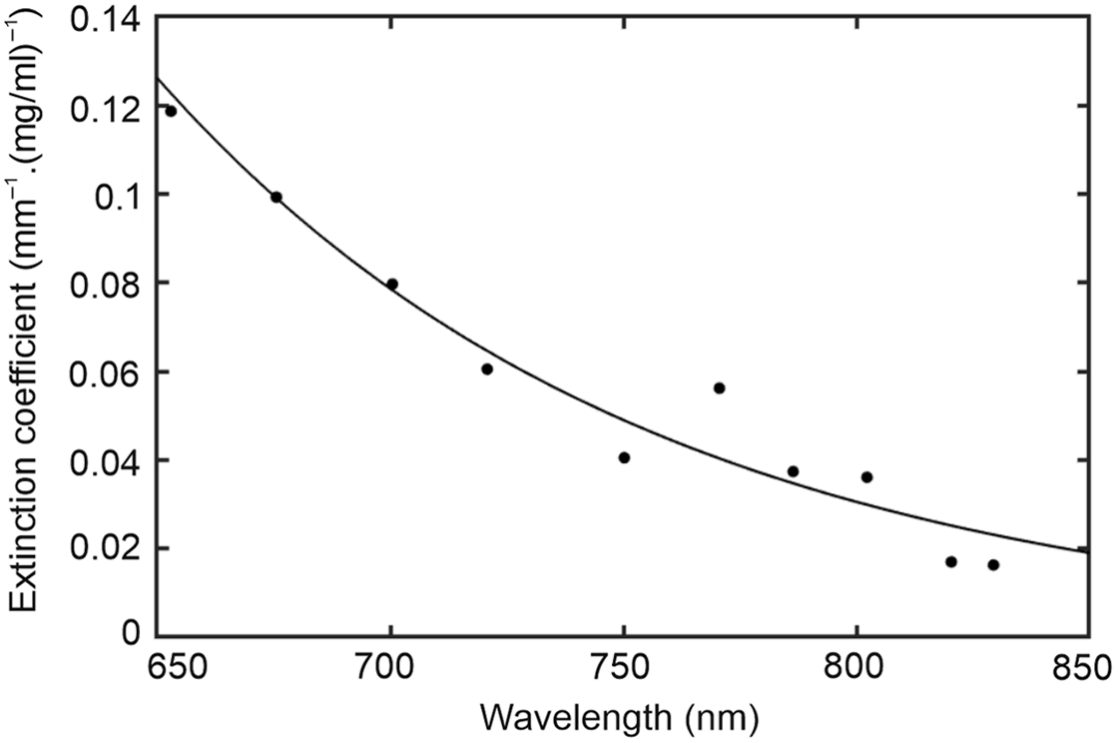
Published measurements of the extinction coefficient of eumelanin (solid circles; from Ref. 16) and an exponential fit to the data (black line).

Optical absorption by black and dark brown hair is predominantly caused by eumelanin, a class of insoluble polymers with optical absorbing characteristics that have been investigated partly as a means of detecting melanoma.^14^ Estimates of the extinction coefficient of eumelanin typically exhibit a consistently smooth and approximately exponential decrease in wavelength over the visible and NIR range of wavelengths. Figure 6 shows the extinction coefficient of eumelanin, expressed in units of ${\mathrm{mm}}^{-1}\cdot (\mathrm{mg}/\mathrm{ml}{)}^{-1}$, as cited by Sarna and Swartz^15^ and available from the online database compiled by Prof. Scott Prahl of the Oregon Institute of Technology.^16^ Figure 6 also shows an exponential fit to these values, over the range 650 to 850 nm.

As described in Sec. 2, Eq. (5) was derived empirically as a means of determining the concentrations of chromophores in homogenous highly scattering media using measurements of the attenuation gradient.^4^ In Secs. 4.2.1 and 4.2.2, this equation is applied to the data acquired on the heterogenous phantom to determine if it can separate the contributions of dye and melanin based on the different wavelength dependencies of their absorption.

#### 4.2.1 Assuming scatter is independent of wavelength

**Fig. 7:**
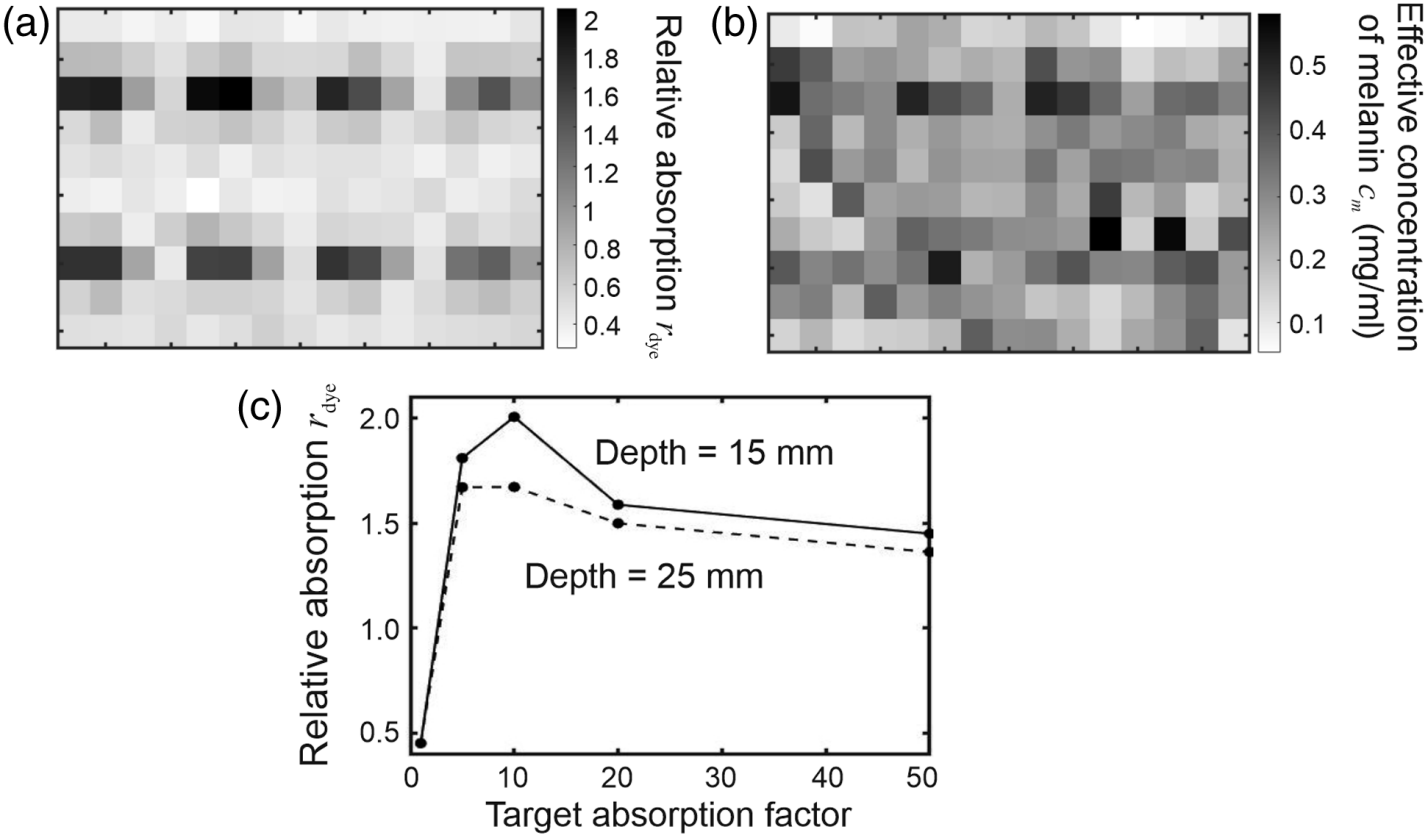
Images of the phantom with the hair layer. (a) Image displaying the variation in the estimation of the absorption produced by the near-infrared dye relative to that of the block; (b) image displaying the variation in the effective concentration coefficient of melanin; (c) derived values of the relative dye absorption at locations closest to each target plotted as a function of target absorption factor.

If the uniform scattering of the phantom is assumed to have no wavelength dependence, then the right-hand term in Eq. (5) can be ignored, and thus, the spectral gradient of the attenuation can be expressed in terms of the gradients of the absorption coefficient of the slab due to the dye $\mu_{a.\text{dye}}$ and the melanin extinction coefficient $\varepsilon_{m}$ as follows:

$$\frac{\partial A}{\partial \lambda }\approx d\langle \beta \rangle (r_{\text{dye}}\frac{\partial \mu_{a,\text{dye}}}{\partial \lambda }+c_{m}\frac{\partial \varepsilon_{m}}{\partial \lambda }),$$ (6)

where $r_{\text{dye}}$ is a unitless measure of relative absorption and $c_{m}$ is an effective melanin concentration in units of mg/ml (effective because the melanin is not distributed within the volume of the phantom as assumed by the equation). The parameter d is the source-detector separation of 20 mm, and the mean DPF is estimated (using a diffusion-based model of a slab with the same optical properties^4^) to have a value of ∼6. Using the measured values of ∂A/∂λ at wavelengths of 760 and 800 nm [shown in Figs. 5(c) and 5(d)] and the estimated derivatives of $\mu_{a,\text{dye}}$ [Fig. 1(c)] and $\varepsilon_{m}$ (from Fig. 6) at the same two wavelengths, values of $r_{\text{dye}}$ and $c_{m}$ were derived for each acquired spectrum by finding the minimum norm least-squares solution to Eq. (6) expressed as a linear matrix equation. The results are displayed in Fig. 7.

The image in Fig. 7(a) represents the spatial variation in $r_{\text{dye}}$, and the absorption due to the dye relative to that of the block exhibits all eight targets with remarkable clarity, although again the highest-absorbing targets are displayed with the lowest contrast. The derived values of $r_{\text{dye}}$ corresponding to the locations closest to each of the eight embedded targets are shown plotted as a function of the target absorption factor in Fig. 7(c). The mean value of the relative absorption within the background region between the rthews of targets is roughly a factor of two lower than the expected value of $r_{\text{dye}}=1$ but shows significantly less random variation than was observed in the gradient images.

The image representing the spatial variation in the effective concentration of melanin $c_{m}$ [Fig. 7(b)] shows a far greater variation within the background regions consistent with a greater sensitivity to the hair layer. The image is qualitatively very similar to the two attenuation images shown in Figs. 5(a) and 5(b), consistent with the expectation that hair is a dominant cause of the attenuation of the diffusely reflected light. Nevertheless, this image also shows some evidence of the embedded targets, indicating that solving Eq. (6) has incompletely separated the effects of the two absorbers. Solving the equation using additional data at other discrete wavelengths over the 700 to 840 nm range was observed to have little influence on either image, either quantitively or qualitatively.

#### 4.2.2 Assuming scatter is dependent on wavelength

Assuming (probably realistically) that the uniform scattering of the phantom has some wavelength dependence, Eq. (5) can be applied as follows:

$$\frac{\partial A}{\partial \lambda }\approx d\langle \beta \rangle (r_{\text{dye}}\frac{\partial \mu_{a,\text{dye}}}{\partial \lambda }+c_{m}\frac{\partial \varepsilon_{m}}{\partial \lambda })-\alpha \lambda^{-p}\;\;.$$ (7)

Solving this equation now requires finding two additional parameters (α and p). Using the same methodology as described in Sec. 4.2.1, images (not shown here) representing spatial variation in the parameters $r_{\text{dye}}$, $c_{m}$, α, and p were generated using measured values of ∂A/∂λ at four or more discrete wavelengths within the range of 700 to 840 nm. Perhaps unsurprisingly, the exponential wavelength characteristics of both $\varepsilon_{m}$ and $\lambda^{-p}$ meant that the influences of melanin and scatter could not be satisfactorily separated, resulting in unrealistic solutions for α and $c_{m}$ (e.g., negative values of $c_{m}$). However, the images representing $r_{\text{dye}}$ remained remarkably robust and varied little, both qualitatively and quantitively, from that shown in Fig. 7(a). It is nevertheless likely that the wavelength dependence of scatter has an influence on the values of $r_{\text{dye}}$, and this may explain why the background regions correspond to a value lower than that expected. But it is also unrealistic to expect the application of Eq. (5) to yield images which are quantitatively accurate. It is essential to note that, when applying the modified Beer–Lambert law [Eq. (1)] to measurements on the phantom containing highly absorbing targets, the parameters β and G will be spatially as well as wavelength dependent, which invalidates the assumptions under which Eq. (5) was derived empirically. One obvious nonquantitative feature of the images is the lower contrast produced by the highest absorbing targets. As described in Sec. 5, Monte Carlo simulations were performed to gain insight into the influence of an embedded target region on the local values of β and G and their derivatives, as well as to confirm the apparent nonlinear relationship between image contrast and target absorption.

## 5 Monte Carlo Modelling

**Fig. 8:**
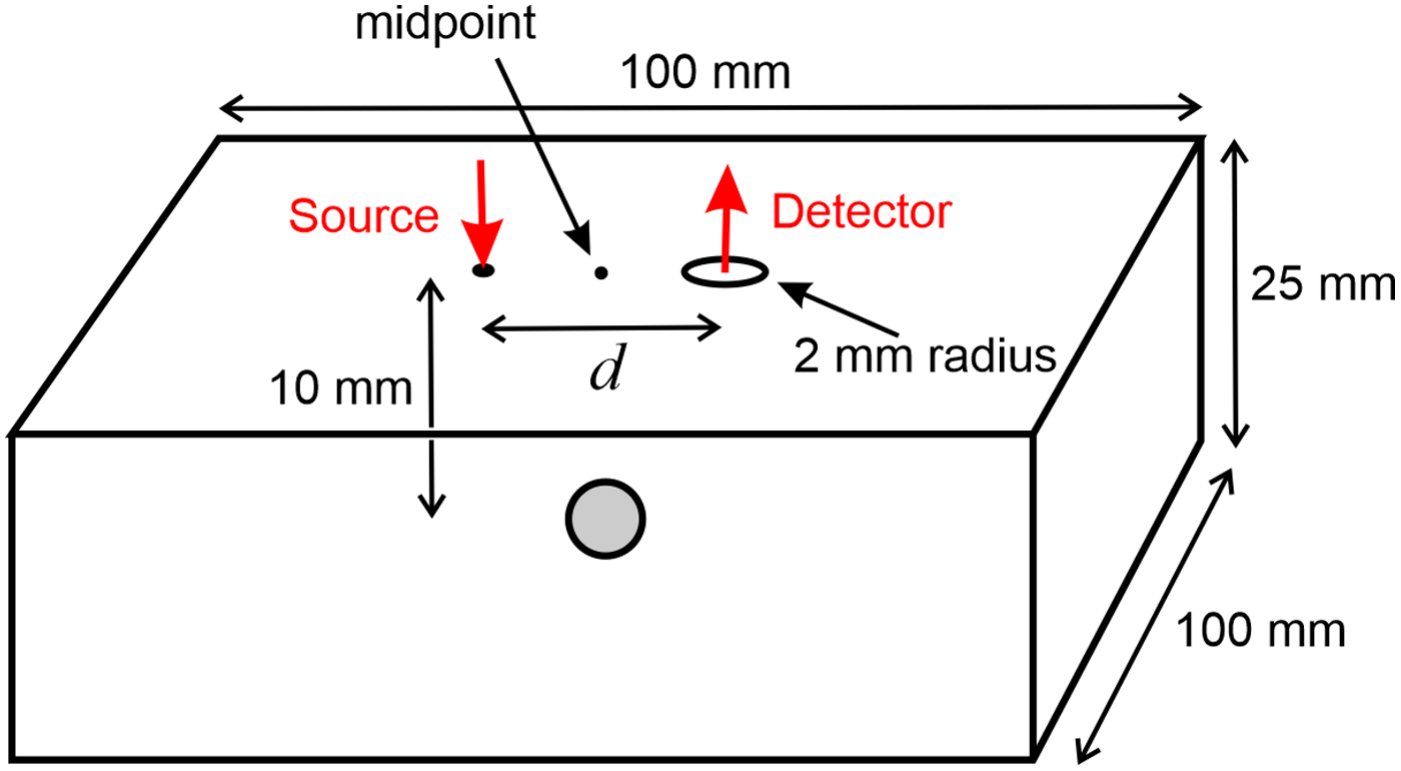
Geometry of a Monte Carlo model of photon migration in a slab containing a single target. The source and detector are separated by d=20  mm. A spherical target of 10-mm diameter is centered at a depth of 10 mm, immediately below the midpoint between the source and detector.

A Monte Carlo model was implemented to simulate measurements on a slab phantom and determine how each of the terms in Eq. (4) varies with wavelength. The model employed the MCX package, a voxel-based open-source light transport simulator funded by the US National Institute of Health.^17^ The model geometry is illustrated in Fig. 8.

A source and detector are separated by 20 mm, either side of the midpoint on the top surface of the slab as shown, with dimensions 100  mm×100  mm×25  mm. Immediately below the midpoint at a depth of 10 mm is a spherical target region of diameter 10 mm. The absorption coefficient of the slab is defined at 19 discrete wavelengths (λ=650,660,670,…,830  nm) at values determined by the absorption spectrum shown in Fig. 1(b). The absorption coefficient of the target is assigned a value of a factor F times that of the surrounding slab, where F is selected at 14 discrete values between 1 and 50. The absorption coefficient of the target was also assigned a value of $1000\;{\mathrm{mm}}^{-1}$ to represent a factor F of infinity (i.e., all incident photons are effectively absorbed by the target). Initially, the same scattering properties are used at each wavelength: a scattering coefficient $\mu_{s}=10\;{\mathrm{mm}}^{-1}$ and a scattering anisotropy parameter g=0.95, so that transport scattering coefficient $\mu_{s}^{\prime }=\mu_{s}(1-g)=0.5\;{\mathrm{mm}}^{-1}$.

**Fig. 9:**
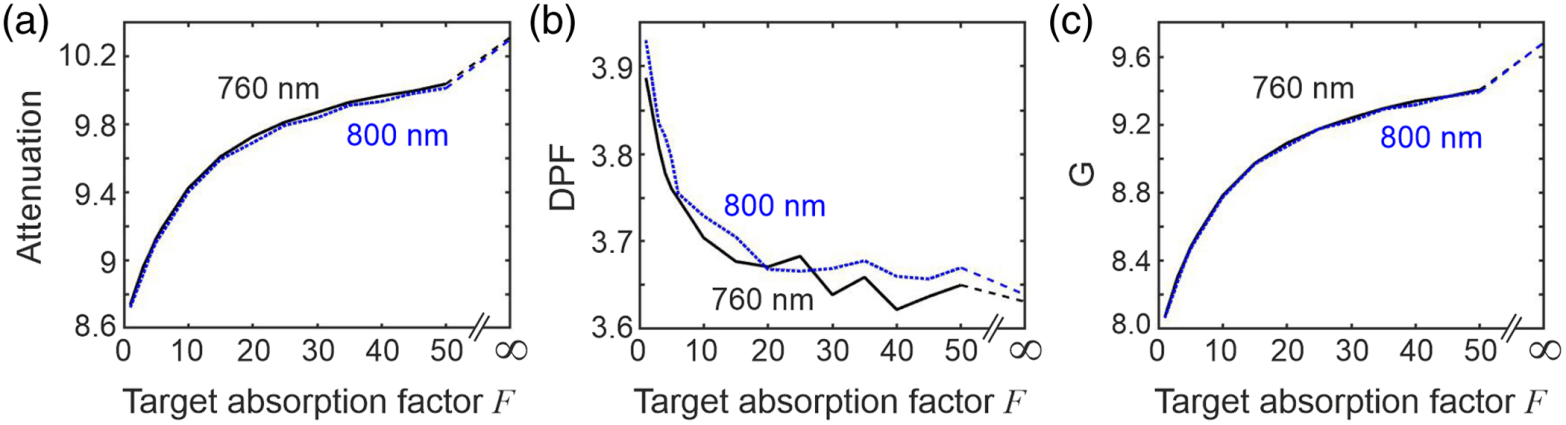
Monte Carlo simulation results for a slab containing a single target. This shows the variations in (a) attenuation, (b) DPF, and (c) G factor at wavelengths of 760 nm (solid black line) and 800 nm (dotted blue line), plotted as a function of the target absorption factor F.

Using $10^{8}$ injected photons, values of attenuation A(λ) and DPF β(λ) were estimated at each of the 16 discrete wavelengths and for each absorption factor F. Because perfect coupling is modeled (i.e., k=1) and $\mu_{a}(\lambda )$ is known, Eq. (3) can be used to determine the geometric factor G(λ) for each case. The three quantities A(λ), β(λ), and G(λ) at wavelengths of 760 and 800 nm are shown plotted against the target absorption factor F in Fig. 9.

The results at 760 and 800 nm are very similar, which is not unexpected given the similar values of the absorption coefficient at these wavelengths: 0.0102 and $0.01\;{\mathrm{mm}}^{-1}$, respectively. Also, as expected, the attenuation A increases with target absorption factor F, although the increase is non-linear for F<20. The DPF decreases by a few percent as the target absorption factor increases from 1 to ∼20 but is then largely unchanged at higher factors. It is interesting to note that the variation in G is highly correlated with that in A, which implies that as target absorption increases, there is a greater loss in detected intensity due to scatter, described by the factor $e^{-G}$.

**Fig. 10:**
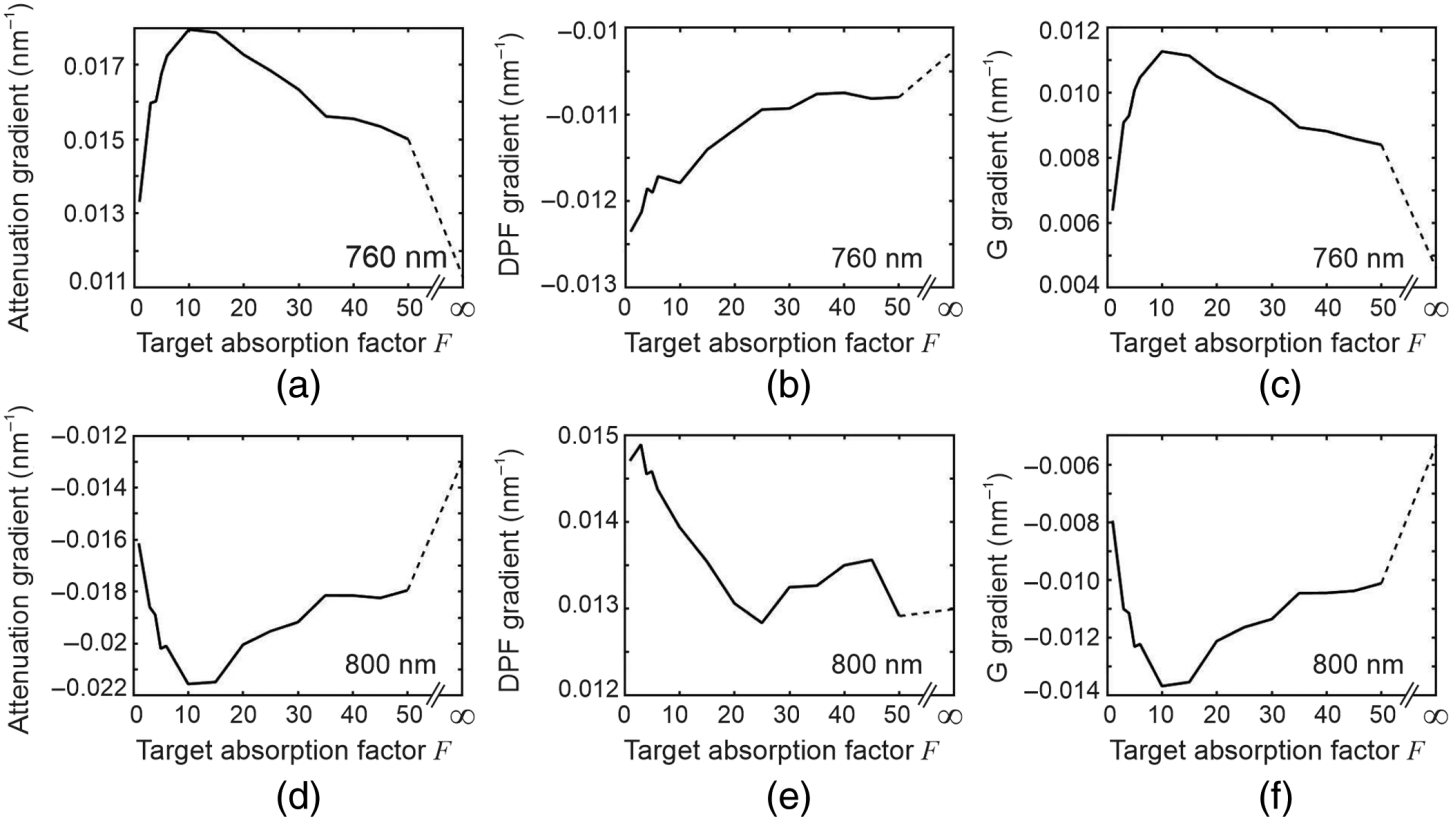
Monte Carlo simulation results for a slab containing a single target. The top row shows the variations in the spectral gradients of (a) attenuation, (b) DPF, and (c) G factor at a wavelength of 760 nm, plotted as a function of the target absorption factor F. In the bottom row, graphs (d), (e), and (f) show the corresponding variations in the spectral gradients of these parameters estimated at a wavelength of 800 nm.

The first derivatives of A(λ), β(λ), and G(λ) with respect to wavelength were then computed by fitting an eighth-order polynomial to each set of 19 values. The estimated derivatives at wavelengths of 760 and 800 nm are shown plotted against the target absorption factor F in Fig. 10.

The attenuation gradient ∂A/∂λ at a wavelength of 760 nm initially increases rapidly with F, exhibits a maximum around F≈10, and then decreases more slowly at higher values. Meanwhile, the attenuation gradient ∂A/∂λ at a wavelength of 800 nm initially decreases with F and also exhibits a minimum around F≈10 before increasing slowly at higher values. Both trends are consistent with the apparent variation in the target contrast observed in the attenuation gradient images at these wavelengths (Fig. 4). Again, the DPF gradients ∂β/∂λ show little variation for F>20, and the variations in ∂G/∂λ are highly correlated with those in ∂A/∂λ.

The modeling was then repeated using a wavelength-dependent scatter of the form: $\mu_{s}=10(\lambda /800{)}^{-1.2}\;{\mathrm{mm}}^{-1}$, where wavelength λ is expressed in nanometers, based on a commonly assumed power relationship.^4^ The results (not shown here) were qualitatively very similar to those shown in Figs. 9 and 10. However, the values of attenuation gradient at both wavelengths were slightly lower by $∼0.002\;{\mathrm{nm}}^{-1}$ at all values of F. The decrease in ∂A/∂λ associated with a negative scatter gradient is consistent with the modeling results observed for homogeneous media reported elsewhere^4^ and is expressed in Eq. (5).

Although there are some minor differences between the physical parameters of the model and those of the experimental phantom (smaller values of $\mu_{s}^{\prime }$ and the target depth were employed to reduce the computation times), the model results confirm the observed nonlinear relationship between target absorption and the contrast achieved in an attenuation gradient image.

The behavior of ∂A/∂λ displayed in Figs. 10(a) and 10(d) may be understood by considering the relative proportion of detected photons that have interacted with the target. When F=1, the target is invisible, and the attenuation gradient ∂A/∂λ will be that of the surrounding medium as expressed by Eq. (6). When F>1 but is still quite small, many of the photons interacting with the target can still reach the detector because the probability of absorption is low. Of those photons, their relative number as a function of wavelength will depend on the wavelength dependence of the target absorption. This causes the initial increase in ∂A/∂λ at 760 nm and the initial decrease at 800 nm. When F increases further, the proportion of detected photons that have interacted with the target decreases due to higher absorption, and ∂A/∂λ increasingly reflects that of photons that *never* interact with the target. Thus, ∂A/∂λ tends to return toward a value closer to that at F=1. When F is very high, virtually every photon interacting with the target is absorbed at every wavelength, and thus, ∂A/∂λ approaches a value at F=∞ that is independent of the absorbing properties of the target. However, the detected photons at F=∞ will have explored a different volume to those detected when F=1. The variation in DPF [Fig. 9(b)] reveals that those detected photons have shorter pathlengths on average (i.e., are mainly propagating above the target), and therefore, the magnitude of the attenuation gradient expressed by Eq. (6) is smaller at F=∞ than at F=1.

Although the highly nonlinear relationship between ∂A/∂λ and target absorption suggests that caution may be needed when interpreting images generated using WM-NIRS, the NIR absorption coefficients of different biological tissues encountered in many practical applications of the method are unlikely to vary by factors exceeding 10,^18^ and therefore, may remain within a relatively linear region [i.e., F<10 in Figs. 10(a) and 10(d)].

## 6 Discussion

The sensitivity of optical measurements to unknown and variable surface coupling has largely limited NIRS and DOT to the assessment of changes in chromophore concentrations and to circumstances in which the movement of the probe relative to the skin is minimal. These are among the factors that have inhibited the clinical translation of these techniques,^19^ and the uncertainty and variability in coupling is inevitably exacerbated by the presence of hair.^8^^,^^20^^,^^21^

The noted insensitivity of spectral derivatives to coupling^9^^–^^11^ offers an attractive means of circumventing or substantially reducing the influence of coupling on DOT images, and the proposed method WM-NIRS, which measures the spectral derivative directly, also offers the potential to derive absolute concentrations of tissue absorbers.^4^ The experiments described in Sec. 3 confirmed that a random distribution of hair has a significantly less deleterious effect on images generated from values of the spectral gradient of the attenuation of the diffusely reflected light than on images generated from more conventional measurements of attenuation (i.e., log intensity). Although attenuation gradient measurements were evidently still influenced by hair, prior knowledge of the (very different) spectral gradients of the absorbing characteristics of the phantom dye and of melanin enabled separate images of each absorber to be acquired. The separation is unlikely to have been so effective if the dye and melanin had had similar spectral gradients.

Although the benefit of using a spectral gradient as an imaging data type is in accordance with expectations, the observed nonlinear relationship between image contrast and target absorption was not anticipated, and the phenomenon potentially limits the imaging performance of the approach. A relatively simple Monte Carlo model was sufficient to confirm and explain the unexpected behavior, and in the future, more sophisticated models could be employed to investigate this further, including those directly incorporating the optical properties of hair.^22^

Although previous experiments have demonstrated the capacity of WM-NIRS to determine absolute values of concentrations of absorbers in homogenous media,^4^ deriving quantitative images of heterogeneous objects is not feasible without adopting a tomographic (DOT) approach using multiple measurements sampling overlapping regions within the object. Attempting image quantitation must also take into account the wavelength dependence of scattering, either as prior information (if known) or as additional parameters to be estimated as part of the reconstruction process [e.g., values of α and p in Eq. (5)]. This also requires ∂A/∂λ to be measured at a number of discrete wavelengths that ideally exceeds the number of fit parameters. A practical implementation must also consider the availability of sources such as laser diodes at the appropriate wavelengths. Dehghani et al.^10^ already successfully demonstrated image reconstruction methods based on simulated spectral derivative data, and a similar “wavelength normalization” approach was developed for time domain data and applied to phantom measurements by Jiang et al.^23^

A desirable next step in the validation of the imaging approach described here is an *in vivo* demonstration on a human subject. It is inevitable that *in vivo* measurements of the attenuation gradient will be sensitive to melanin content in skin as well as in hair, but it should be feasible to separate its influence from those of ${\mathrm{HbO}}_{2}$, Hb, and other chromophores. Acquiring a series of measurements at a large number of discrete locations on the scalp to generate a static image of the brain is prohibitively time consuming using a single spectrometer. Instead, an imaging system suitable for human subjects needs to acquire data in parallel using multiple sources and detectors. For example, a system based on pairs of laser diodes emitting at adjacent wavelengths (i.e., a few nanometers apart) has been proposed.^3^

Finally, it must be noted that the presence of hair is not the only cause of variability in surface coupling for NIRS and DOT measurements. Another potential cause is perspiration between the probe and the skin, which would likely cause a local change in reflectivity, which may have some wavelength dependence. Probe movement may also produce pressure changes resulting in a localized change in blood volume within microvessels directly below the surface.^8^ It is unlikely that a WM-NIRS approach would be immune to such changes.

## 7 Conclusions

Variable coupling due to hair is a major factor in inhibiting imaging of the static optical properties of biological tissues and of the brain in particular. The potential of a simple method of generating topographic maps of tissue-like media using measurements of the attenuation gradient was demonstrated based on the modified Beer–Lambert law. The results confirmed that spectral derivative data can eliminate the effects of hair from static images, but only if the spectral dependence of melanin absorption by hair is accounted for. It was also concluded that the relationship between the measured attenuation gradient and the intrinsic absorption of embedded features is highly nonlinear when their absorption is large, suggesting that images generated using spectral derivatives must be interpreted with caution.

## Data Availability

Data underlying the results presented in this paper are not publicly available at this time but may be obtained from the authors upon reasonable request.
